# Thermodynamic Analysis of Irreversible Desiccant Systems

**DOI:** 10.3390/e20080595

**Published:** 2018-08-09

**Authors:** Niccolò Giannetti, Seiichi Yamaguchi, Andrea Rocchetti, Kiyoshi Saito

**Affiliations:** 1 Interdisciplinary Institute for Thermal Energy Conversion Engineering and Mathematics, Waseda University, Shinjuku-ku, Tokyo 169-8555, Japan; 2 DIEF—Department of Industrial Engineering of Florence, University of Florence, Via Santa Marta 3, 50139 Firenze, Italy

**Keywords:** thermodynamic mapping, desiccant systems, entropy balance

## Abstract

A new general thermodynamic mapping of desiccant systems’ performance is conducted to estimate the potentiality and determine the proper application field of the technology. This targets certain room conditions and given outdoor temperature and humidity prior to the selection of the specific desiccant material and technical details of the system configuration. This allows the choice of the operative state of the system to be independent from the limitations of the specific design and working fluid. An expression of the entropy balance suitable for describing the operability of a desiccant system at steady state is obtained by applying a control volume approach, defining sensible and latent effectiveness parameters, and assuming ideal gas behaviour of the air-vapour mixture. This formulation, together with mass and energy balances, is used to conduct a general screening of the system performance. The theoretical advantage and limitation of desiccant dehumidification air conditioning, maximum efficiency for given conditions constraints, least irreversible configuration for a given operative target, and characteristics of the system for a target efficiency can be obtained from this thermodynamic mapping. Once the thermo-physical properties and the thermodynamic equilibrium relationship of the liquid desiccant mixture or solid coating material are known, this method can be applied to a specific technical case to select the most appropriate working medium and guide the specific system design to achieve the target performance.

## 1. Introduction

In recent years, increasing attention has been paid to enthalpy recovery, in which both the sensible and the latent heat are involved. Devices which simultaneously transfer heat and mass are commonly used in the power and Heating, Ventilation, Air Conditioning, and Refrigeration (HVAC&R) systems to control the temperature and humidity of a conditioned space. Frequently, the latent load of a conditioned room constitutes the largest fraction of the total thermal load (Abdel-Salam and Simonson [1]); hence, it is substantial to rationalise the management of latent as well as sensible heat transfer. Conventional air-conditioning systems cope with this issue inefficiently, dehumidifying by lowering the moist air temperature below its dew point (see, for instance, the comparative work of Pesaran [2]). Sorption processes and desiccant materials represent an alternative approach, which enables the system to achieve the desired air dehumidification rate directly, suspending the constraint of reaching the dew point of the inlet air stream, and with several other attractive features. Typically, the system performs an open isobaric cycle where the refrigerant is air and hence easy to handle; free; ubiquitously available; and harmless to users, operators, and environment. Since the energy rejection and absorption occurs directly the transfer irreversibility can be substantially lowered (i.e., the temperature of the air entering a conditioned room can be relatively high). Low exergetic level heat (60–90 °C) (Tu et al. [3]), rather than electricity, can be used as the main energy source, enabling the use of renewable sources (Jani et al. [4]) and waste heat recovery (Ge et al. [5]). A higher system reliability (Giampieri et al. [6]) can be ascribed to the absence of a compressor. Desiccant-cooling systems employ a sorptive material that undergoes a cycle of thermodynamic transformations by sequentially facing the process air and the regeneration stream, thus realising the necessary heat and mass exchanges. These processes are driven by the characteristic vapour pressure at the desiccant-air interface, which is related to the equilibrium isotherm of the specific desiccant material: a univocal relation between the moisture content, pressure and temperature. During the sorption of moisture, the related heat of sorption is released and transmitted through the material itself, which decreases its sorption capacity. Therefore, heat and mass transfer are coupled processes influenced by the contactor configuration, the thermo-physical properties of the desiccant material, the fluid dynamics of the air streams and the contingent operating conditions. Furthermore, a large variety of desiccant mixtures and coatings are available and more new materials are under development. For an updated literature review about absorptive or adsorptive materials and desiccant cycle configurations refer to Mujahid-Rafique et al. [7], Fu and Liu [8], and Wu et al. [9], respectively. In this respect, the development of Ionic Liquids (IL) is a subject currently of great interest and constitutes one of the most promising options. Ionic Liquids are molten salts, in the common range of operative temperatures, consisting of organic cations and inorganic anions, which have several advantages when compared to conventional working fluids (i.e., aqueous Lithium-cloride). Besides being generally non-flammable, having good chemical and thermal stability, large chemical energy storage potential, and dust and bacteria removal ability, by loosening some of the technological constraints of LiCl-water solution, such as crystallisation and corrosion toward metallic structures, expound the applicability of this technology (Deng [10]). Luo et al. [11] presented the feasibility of [Emim]BF_4_ as an IL desiccant solution, and in a following study, Luo et al. [12] tested the surface vapour pressures of aqueous solutions of [Bmim]BF_4_ and [Dmim]OAc, finally comparing the dehumidification performance of the latter with conventional desiccants. A similar comparison was carried out by Zegenhagen et al. [13], whereas, Varela et al. [14] and Giannetti et al. [15], respectively, studied and modelled the wetting ability of an IL solution at different mass fractions flowing over the aluminium substrate of a newly designed internally cooled/heated contactor.

Potentially, there are up to 10^18^ different kinds of Ionic Liquids, as a result of the combination of different anions and cations. This opens up to new possibilities in terms of optimised system design, where not only the structure is adapted to load, condition, and volume constraints, but also the most suitable working fluid development can contribute in maximising the transfer performance for achieving energy savings of higher orders of magnitude. This technical progress demands for a renovated theoretical basis able to cover the enlarged applicability field of this technology.

Due to the complexity and nonlinearity of the governing differential equations characterising the energy and mass conservation laws, numerical (Yamaguchi and Saito [16]) and experimental (Varela et al. [17]) methods have been often applied in modelling such systems, or the attention has been directed towards one specific aspect of the process (Giannetti et al. [18]).

Notwithstanding the broad interest and intensive research attracted by this technology, to the authors’ knowledge, a common general theoretical basis for the design of desiccant systems has not been conclusively established yet. Commonly, modelling efforts of the related transport performance follows (see, for instance, Ge et a. [19], Jeong et al. [20] or Yamaguchi et al. [21]) the selection of a specific desiccant material and a defined structure of the contactors (heat and mass exchangers); hence, limiting the generality of the modelling results, and consequently, the degree of freedom for achieving optimised solutions. To assess the potentiality of a certain technology in a preliminary design stage, it is required to be able to predict the system performance and its limitations under a broad range of possible operative conditions. For the sake of generality, technical details of a certain system, which include the designated type of desiccant, should not be fixed prior the analysis, but selected (or designed) to operate as close as possible to the highest technical limitations of the system (Giannetti et al. [22,23]).

The formulation of performance evaluation methods and optimisation criteria has often accompanied the study of thermal engineering systems. Although desiccant processes are characterised by a higher degree of complexity when writing the governing transport laws and conservation principles, their theoretical background is still based on the classical laws of thermodynamics. To achieve a comprehensive and general evaluation approach, first and second laws of Thermodynamics along with the mass balances are to be assessed. Regarding dehumidification systems, following the pioneering work of Lavan et al. [24] which established the reversible upper bounds of an isobaric open desiccant cooling cycle independently from the nature of the desiccant material and introduced the definition of equivalent source and sink temperatures, finite time Thermodynamics has played an important role, although in a limited number of examples, in moving the attention towards new irreversibility sources. Pons and Koyama [25] has performed a second principle analysis on the internal and external irreversibility contributions of an open ventilation cycle. Konoglu et al. [26] prepared the effects of ambient conditions in terms of individual exergy losses within the cycle’s components. More recently, La et al. [27] treated and analysed each source of irreversibility to greater detail.

To contribute to the establishment of a common theoretical basis of desiccant systems, independently of the thermo-physical properties of the working medium, and from this fundamental standpoint assess their technical potential providing a shared term of comparison with different technologies and extracting universal guidelines for actual system design and control, a general thermodynamic analysis is performed. The development of a new expression of the entropy balance suitable for describing the operability of a desiccant system at steady state includes the irreversibility related to heat and mass transfer phenomena, and deepens Laval’s work [24] while maintaining its generality. An expression of the entropy balance, suitable for describing the operability of a desiccant system at steady state, is obtained independently from the type of desiccant employed by applying a control volume approach, defining sensible and latent effectiveness parameters, and assuming ideal gas behaviour of the air-vapour mixture. The impact of the operating conditions and internal system irreversibility is emphasised delineating a suitable application field. The maximum efficiency for given conditions constraints, the least irreversible configuration for a given operative target, as well as the characteristics of the system for a target efficiency can be obtained. Once the thermo-physical properties and the thermodynamic equilibrium relationship of the desiccant mixture are known, this method can be applied to a specific technical case to select the most appropriate working fluid and guide the specific system design. Finally, a graphical analysis, based on data from literature, is carried out to illustrate the use of this thermodynamic criterion for desiccant systems.

## 2. Modelling

**Figure 1:**
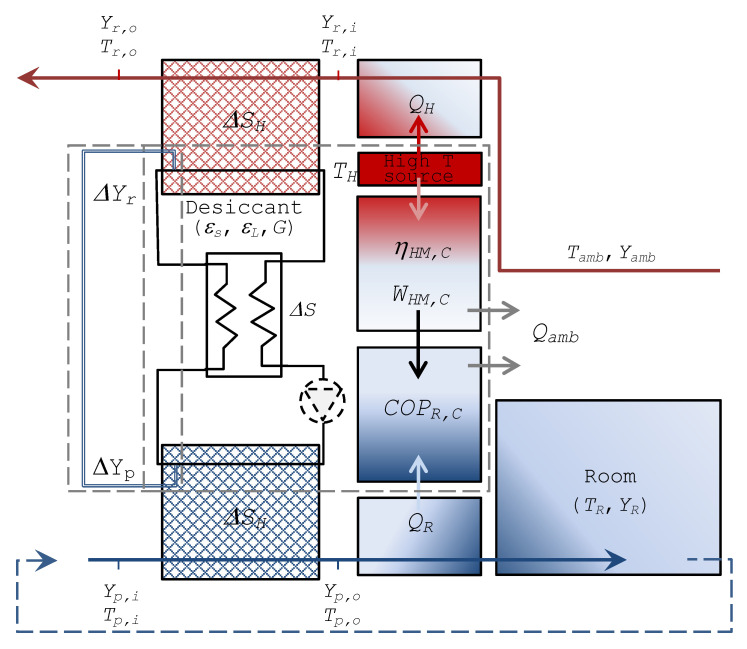
Schematic of the desiccant-cooling system under consideration.

A schematic representation of the system under consideration is presented in Figure 1. To maintain a conditioned room at targeted indoor conditions a desiccant cooling system is used. To achieve the desired indoor absolute humidity *Y_p,i_* = *Y_R_*, the process-air from the outdoor ambient *T_amb_*, or at an intermediate temperature (in case of recirculation and mixing) and inlet absolute humidity *Y_p,i_* faces an adiabatic dehumidification process, which is associated to higher outlet temperatures *T_p,o_*. Subsequently, the air stream is cooled down to the required indoor temperature *T_R_* and delivered to the conditioned room. For this latter process a dedicated chiller working between the outlet process air temperature level *T_p,o_* and *T_R_* can be generally used, but for most of the air-conditioning applications the scale of the temperature jump is small enough to be realised by indirect evaporative cooling or a Maisotsenko cycle (Caliskan et al. [28]), hence, opening up to the possibility of a desiccant-cooling system (Saghafifar and Gadalla [29]; Pandelidis et al. [30]) that works exclusively with natural refrigerants.

On the regeneration side of the desiccant system, high temperature air (*T_r,i_*) with low relative humidity is used to extract the absorbed amount of vapour from the sorptive medium and steadily repeat the cycle.

### 2.1. Thermal Cycle Efficiency

Let us consider an isobaric (*P_amb_* = *P_a_*) liquid desiccant system operating at steady state, where the working fluid steadily complete a cycle. Imagining that the absorbed vapour on the process side is used to make up for the desorbed vapour on the regeneration side, the inclusion of this internal transport process closes the cycle. In this case, the entropy variation of the sorptive solution is null (initial state = final state). Thus, the internal irreversibility Δ*S* should be transferred outside the cycle through heat exchangers/contactors. La et al. [27] correspondingly introduced virtual condenser and evaporator in a solid desiccant ventilation cycle to analyse it in the same way as conventional closed thermodynamics cycles. Similarly, Pons and Kodama [25] considered virtual systems that would “close” an adsorptive cooling cycle, while Lavan et al. [24] alternatively introduced the concept of “entropic mean temperature”.

From this standpoint, the reversible thermodynamic structure of the desiccant ventilation cycle is similar to the three-thermal cooling cycle, and it can be conceived as the combination of a heat engine and a refrigerator.

(1) $$COP_{C}=\eta_{HM,C}COP_{R,C}=\left(1-\frac{T_{amb}}{T_{H}}\right)\left(\frac{T_{R}}{T_{amb}-T_{R}}\right)$$

When considering the first law of Thermodynamics, Equation (2) is written.

(2) $$Q_{H}+Q_{R}+Q_{amb}=0$$

The second law of Thermodynamics is expressed by Equation (3).

(3) $$\frac{Q_{H}}{T_{H}}+\frac{Q_{R}}{T_{R}}+\frac{Q_{amb}}{T_{amb}}+\Delta S=0$$

Finally, the thermal efficiency of the system *η_TH_* can be written as in Equation (4) (see, for instance La et al. [27]).

(4) $$\eta_{TH}=\frac{Q_{R}}{Q_{H}}=\frac{1-\frac{T_{amb}}{T_{H}}}{\frac{T_{amb}}{T_{R}}-1}\left[1-\frac{T_{amb}\Delta S}{Q_{H}\left(1-\frac{T_{amb}}{T_{H}}\right)}\right]=COP_{C}\left[1-\frac{T_{amb}\Delta S}{Q_{H}\left(1-\frac{T_{amb}}{T_{H}}\right)}\right]$$

### 2.2. Energy and Mass Balance

This formulation relates temperature and absolute humidity of process and regeneration streams through the definition of sensible and latent effectiveness (Equations (5) and (6)). For constant, or process-averaged, specific heat, the sensible effectiveness *ε_s_* is given by,

(5) $$\varepsilon_{s}=\frac{\overset{•}{m_{d,p}}c_{pd,p}}{{\overset{•}{(m}}_{d}c_{pd}{)}_{\min}}\frac{\left(T_{p,o}-T_{p,i}\right)}{\left(T_{r,i}-T_{p,i}\right)}=\frac{\overset{•}{m_{d,r}}c_{pd,r}}{{\overset{•}{(m}}_{d}c_{pd}{)}_{\min}}\frac{\left(T_{r,i}-T_{r,o}\right)}{\left(T_{r,i}-T_{p,i}\right)}$$

Whereas, the latent effectiveness *ε_L_* is expressed as in Equation (6).

(6) $$\varepsilon_{L}=\frac{\overset{•}{m_{d,p}}}{{\overset{•}{(m}}_{d}{)}_{\min}}\frac{\left(Y_{p,i}-Y_{p,o}\right)}{\left(Y_{p,i}-Y_{r,i}\right)}=\frac{\overset{•}{m_{d,r}}}{{\overset{•}{(m}}_{d}{)}_{\min}}\frac{\left(Y_{r,o}-Y_{r,i}\right)}{\left(Y_{p,i}-Y_{r,i}\right)}$$

### 2.3. Entropy Balance

Being entropy a state function, in a closed thermodynamic cycle the entropy variation of the working fluid is null. Accordingly, if heat and sorptive solution mass leakages are overlooked, the internal irreversibility (pressure drops, heat transfer, mass transfer, mixing, etc.) is transferred to the regeneration and process air-streams through the desiccant contactors (dehumidifier and regenerator). In general, considering a control volume that encloses the desiccant working cycle and the contactors, and referring to the specific entropy of air as a mixture of perfect gas components (dry air and vapour content), Equation (7) can be written; this can be developed referring to the dimensionless parameters listed in Table 1.

(7) $$\begin{array}{l} \frac{\Delta S}{{\overset{•}{m}}_{d,p}}=c_{pd,p}\ln\left(\frac{T_{p,o}}{T_{p,i}}\right)-R_{d}\ln\left(\frac{p_{dp,o}}{p_{dp,i}}\right)+\frac{1}{\mu }c_{pd,r}\ln\left(\frac{T_{r,o}}{T_{r,i}}\right)-R_{d}\frac{1}{\mu }\ln\left(\frac{p_{dr,o}}{p_{dr,i}}\right)\\ +Y_{p,o}\left[c_{pv,p}\ln\left(T_{p,o}\right)-R_{v}\ln\left(p_{vp,o}\right)\right]-Y_{p,i}\left[c_{pv,p}\ln\left(T_{p,i}\right)-R_{v}\ln\left(p_{vp,i}\right)\right]\\ +\frac{Y_{r,o}}{\mu }\left[c_{pv,r}\ln\left(T_{r,o}\right)-R_{v}\ln\left(p_{vr,o}\right)\right]-\frac{Y_{r,i}}{\mu }\left[c_{pv,r}\ln\left(T_{r,i}\right)-R_{v}\ln\left(p_{vr,i}\right)\right] \end{array}$$

Developing the partial pressure ratios (negative pressure drop Δ*p* is considered),

(8) $$\frac{p_{d,o}}{p_{d,i}}=\frac{p_{a}+\Delta p-p_{v,o}}{p_{a}-p_{v,i}}=\frac{0.622+Y_{i}}{0.622+Y_{o}}\left[1+\frac{\Delta p}{p_{a}}\left(1+\frac{Y_{o}}{0.622}\right)\right]\;$$

Typical operative conditions of an air conditioning application enable the algebraic approximation of Equation (9) (Δ*p*/*p_a_* << 1), and the numerical negligibility of the terms with the shape of Equation (10).

(9) $$\ln\left(1+\frac{\Delta p}{p_{a}}\right)\approx \frac{\Delta p}{p_{a}}$$

(10) $$Y\frac{\Delta p}{p_{a}}\approx 0$$

Additionally, it is assumed

(11) $$\frac{Y}{0.622+Y}≅\frac{Y}{0.622}$$

A relatively handling analytical expression can be finally obtained by neglecting dry air partial pressure variations and treating partial vapour pressure ratios as the corresponding absolute humidity ratios. This implies that, in a range of absolute humidity *Y* < 0.04 kg/kg*_a_*, the relative error is limited below 6.5% (Giannetti et al. [31]).

(12) $$\frac{0.622+Y_{o}}{0.622+Y_{i}}=1$$

Therefore, Equation (13) is obtained.

(13) $$\begin{array}{l} G=\frac{s_{gen}}{c_{pd,p}}=\frac{\Delta S_{gen}}{c_{pd,p}{\overset{•}{m}}_{d,p}}=\frac{Y_{p,i}\varepsilon_{L}\left(\chi -1\right)}{z_{M}}\left[z_{M}\left(1-\frac{1}{\gamma_{v}}\right)\ln\left(\chi \right)+\frac{1}{z_{v}}R_{v}\ln\left(T_{r,i}\right)\right]\\ -\left(1-\frac{1}{\gamma_{d}}\right)\left(\frac{\Delta p_{p}}{p_{a}}+\frac{1}{\mu }\frac{\Delta p_{r}}{p_{a}}\right)+\left\{1+Y_{p,i}z_{M}\left[1+\varepsilon_{L}\left(\chi -1\right)\right]\right\}\ln\left(T_{p,o}\right)\\ -\ln\left(\frac{T_{p,o}-\varepsilon_{s}T_{r,i}}{1-\varepsilon_{s}}\right)+\frac{1}{\mu }\left\{\frac{1}{z}+Y_{p,i}\frac{z_{M}}{z_{v}}\left[\mu z\varepsilon_{L}\left(1-\chi \right)+\chi \right]\right\}\\ \times \left\{\ln\left[T_{r,i}-\mu zT_{p,o}+\mu z\left(\frac{T_{p,o}-\varepsilon_{s}T_{r,i}}{1-\varepsilon_{s}}\right)\right]-\ln\left(T_{r,i}\right)\right\}-Y_{p,i}z_{M}\left(1-\frac{1}{\gamma_{v}}\right)\\ \times \{\left[1+\varepsilon_{L}\left(\chi -1\right)\right]\ln\left[1+\varepsilon_{L}\left(\chi -1\right)\right]+\left[\frac{\mu z\varepsilon_{L}\left(1-\chi \right)+\chi }{\mu }\right]\ln\left[\frac{\mu z\varepsilon_{L}\left(1-\chi \right)+\chi }{\chi }\right]\} \end{array}$$

## 3. Thermodynamic Analysis

Energy, mass, and entropy balances (Equations (5)–(13)) enable the investigation of the performance of this technology in different conditions with reference to the thermal efficiency, as developed in Equation (14).

(14) $$\eta_{TH}=\frac{1-\frac{T_{amb}}{T_{r,i}}}{\frac{T_{amb}\left(1-\varepsilon_{s}\right)}{\left(T_{p,o}-\varepsilon_{s}T_{r,i}\right)}-1}\left[1-\frac{T_{amb}G}{\left(\frac{1}{z}+Y_{r,i}\frac{z_{M}}{z_{v}}\right)\frac{{\left(T_{r,i}-T_{amb}\right)}^{2}}{T_{r,i}}}\right]$$

**Figure 2:**
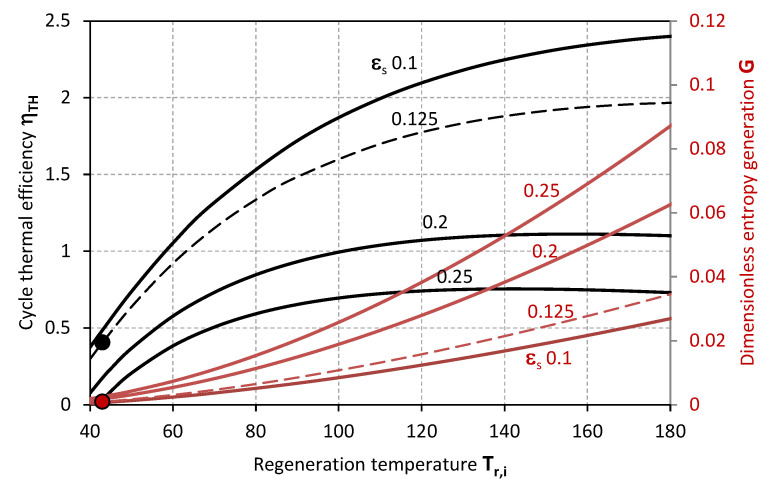
Thermal efficiency (black lines) and dimensionless entropy generation (red lines) as functions of the inlet air regeneration temperature for different values of thermal effectiveness.

**Figure 3:**
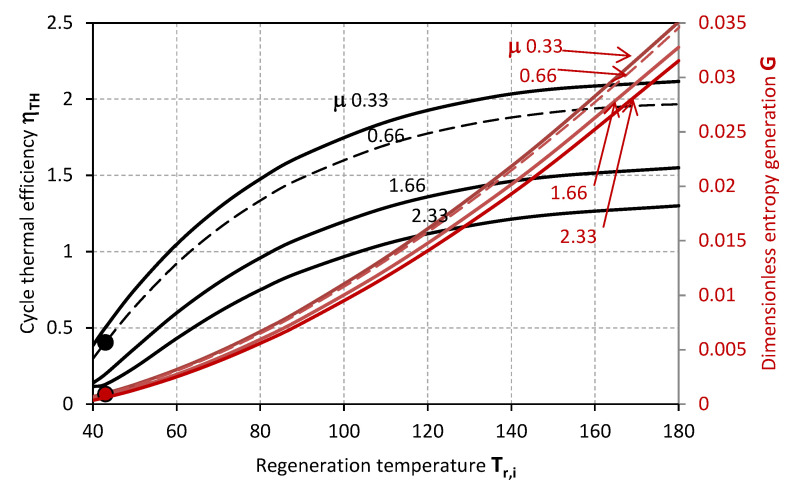
Thermal efficiency (black lines) and dimensionless entropy generation (red lines) as functions of the inlet air regeneration temperature for different values of the ratio of process to regeneration flow rates.

**Figure 4:**
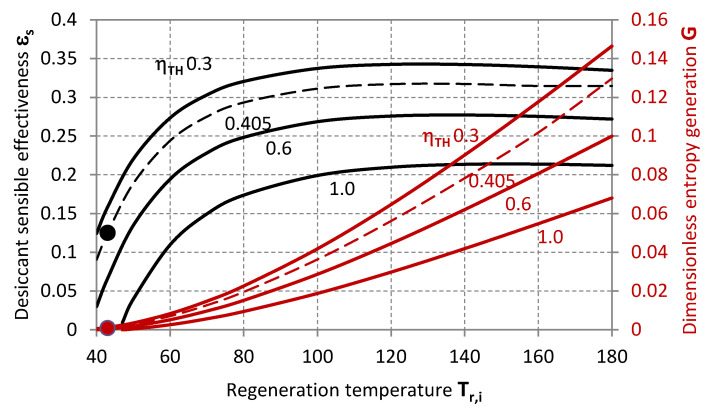
Sensible effectiveness (black lines, primary axis) and dimensionless entropy generation (red lines, secondary axis) as functions of the inlet air regeneration temperature for different target values of the thermal efficiency of the cycle.

**Figure 5:**
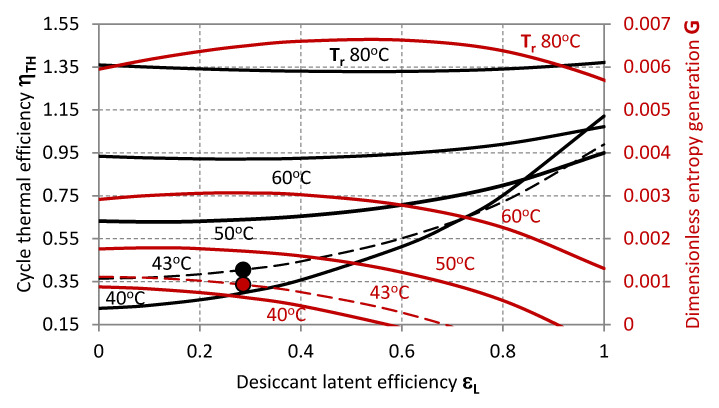
Thermal efficiency (black lines, primary axis) and dimensionless entropy generation (red lines, secondary axis) as functions of the latent effectiveness for different values of the inlet regeneration temperature.

**Figure 6:**
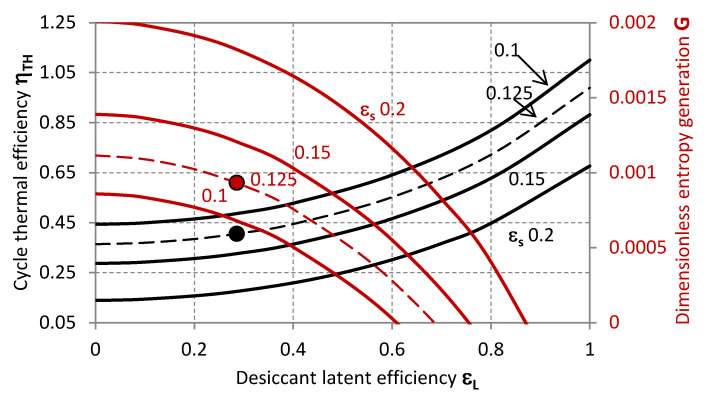
Thermal efficiency (black lines, primary axis) and dimensionless entropy generation (red lines, secondary axis) as functions of the latent effectiveness for different values of the thermal effectiveness.

**Figure 7:**
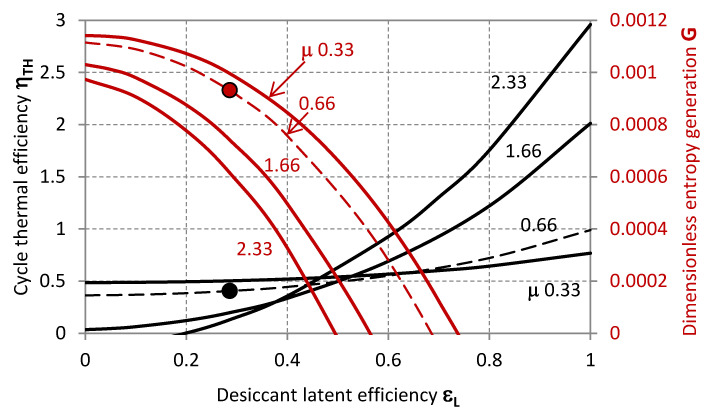
Thermal efficiency (black lines, primary axis) and dimensionless entropy generation (red lines, secondary axis) as functions of the latent effectiveness for different values of the ratio of the process to the regeneration flow rates.

**Figure 8:**
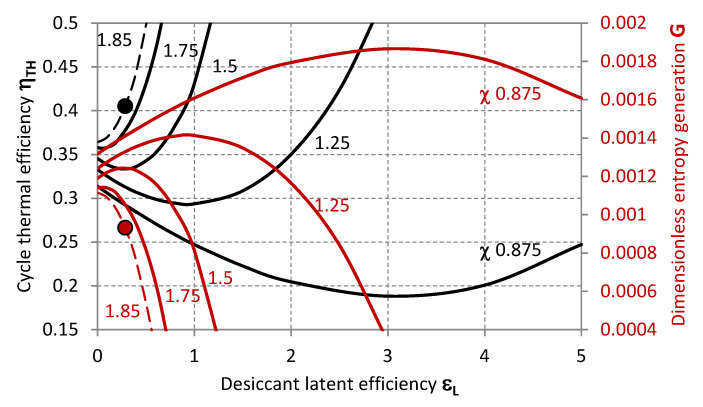
Thermal efficiency (black lines, primary axis) and dimensionless entropy generation (red lines, secondary axis) as functions of the latent effectiveness for different values of the ratio of the absolute humidity of the process to the regeneration air-stream.

The operative state of the system experimentally characterised by Mohan et al. [32] is summarised in Table 2 and plotted within the characteristic graphs in Figure 2, Figure 3, Figure 4, Figure 5, Figure 6, Figure 7 and Figure 8 as a reference state from the standpoint of which the system characteristics, behaviour in terms of external disturbances, or possible cycle improvements are evaluated.

Figure 2 and Figure 3 make evidence for the effect of *T_r,i_* on the thermal efficiency of the desiccant cooling cycle *η_TH_* and the dimensionless entropy generation *G* when sensible effectiveness *ε_s_* and the ratio of process and regeneration air flow rates *μ* are varied. Specifically, the thermal efficiency of the cycle *η_TH_* decreases along with the sensible effectiveness *ε_s_* and higher values of *μ*, whereas *G* inversely increases with *ε_s_*, and decreases with *μ*. On the one hand, a maximal efficiency can be identified for a certain inlet temperature of the regeneration air stream *T_r,i_*; this latter, moves to lower values as *ε_s_* increases. On the other hand, the dimensionless entropy generation *G* relentlessly increases as *T_r,i_* grows higher.

The markers represent the actual operative condition of the desiccant system referenced in Table 2 [32], highlighting, on the one hand, the necessity of higher regeneration temperatures for achieving higher thermal performance of the cycle. However, this is associated to higher entropy generation rates *G*, and, eventually, an excessive increase of the inlet temperature of the regeneration air-stream *T_r,i_* could also affect the sensible efficiency having a detrimental effect on the thermal efficiency of the cycle *η_TH_*. The experimental operability of the system from Mohan et al. [32] exhibits limited cycle thermal performance, which can be related to the range of operative temperatures limited by the mixture stability or the modest transport performance of the specific configuration of the contactors. Specifically, the steady operability of the system is associated to a cycle thermal efficiency of 0.405. This value is in noteworthy accordance with the thermal performance experimentally analysed by Mohan et al. [32]. Nonetheless, higher cycle efficiency values can be achieved, as previously mentioned, provided that the desiccant material is compatible with higher regeneration temperatures, and the component structure with lower flow rate ratio *μ* (Figure 3), lower sensible effectiveness *ε_s_* (Figure 2) and higher latent effectiveness *ε_L_* (Figure 5, Figure 6 and Figure 7).

Figure 4 illustrates the required values of *ε_s_* and *G*, for achieving a certain thermal efficiency of the cycle with required dehumidification rate (given *ε_L_*). These quantities are related to the configuration of the contactor, the thermo-physical properties of the working fluid or the adsorptive coating material and the specific operative conditions, including the relative balance characteristics in terms of heat capacity and humidity contents of the two air-streams (*χ*, *μ*).

Figure 5, Figure 6, Figure 7 and Figure 8 illustrate further characteristics of the system as the dehumidification rate is varied for different values of *χ*, *μ*, *ε_s_* and *T_r,i_*, highlighting (markers) the operative state of the desiccant system referenced in Table 2 and indicating the shifting of the operative condition to higher *χ*, *ε_s_* and *T_r,i_*, or lower *ε_s_* and *μ*.

More specifically, Figure 5 plots *η_TH_* and *G* as functions of *ε_L_*, showing that higher dehumidification rates are thermally beneficial at low regeneration temperatures *T_r,i_*, but less influent at higher *T_r,i_*. A minimal thermal efficiency *η_TH_* appears for inlet regeneration temperatures higher than 50 °C, and corresponds to a maximal value of *G*. These are associated to growing *ε_L_* when *T_r,i_* is increased.

In Figure 6 *η_TH_* and *G* are analysed with respect to *ε_L_* when the sensible efficiency *ε_s_* is varied. For a correct interpretation of this graph, the fact that, as the working fluid is selected or the contactor designed, these two parameters cannot be varied independently should be contemplated; namely, higher dehumidification rates are usually associated to higher *ε_s_*.

The effect of *μ* on *η_TH_* and *G* can be summarised as follows: low *ε_L_* operation corresponds to a moderately beneficial effect of lower *μ* on the thermal efficiency of the cycle; ceteris paribus, higher *μ* positively affect *η_TH_* at higher *ε_L_*.

When *χ* (humidity ratio at the inlet of the air-streams) is varied, the thermal performance of the cycle is illustrated in Figure 6 as a function of *ε_L_*. It can be observed that, depending on the target nominal condition of the system to be designed, the operative range of *ε_L_* should be adapted to avoid minimal or decreasing *η_TH_*, which correspond to maximal or increasing *G*. The value of *ε_L_* matching the minimal *η_TH_* (and maximal *G*) moves to higher values as the absolute humidity at the inlet of the regenerator *Y_r,i_* increases for fixed *Y_p,i_*.

## 4. Conclusions

A general thermodynamic analysis of desiccant cooling systems is proposed to investigate the potentiality and characteristics of this technology with reference to the main influent parameters, obtaining a general performance map covering a wide range of operative conditions. Additionally, the guidelines extracted from a first screening of the results were referred to as the actual operation of a system from previous literature by placing its state within the developed performance map. This has led to the following main conclusions:

- A maximal efficiency can be identified for a certain inlet temperature of the regeneration air stream *T_r,i_*, which moves to lower values as *ε_s_* increases;
- Ceteris paribus, at operative conditions of low *ε_L_* working with higher ratios of the process to the regeneration flow rates *μ*, is detrimental to the thermal efficiency of the cycle, whereas the opposite can be generally advised at *ε_L_* higher than 0.5;
- A maximal entropy generation per unit dry air stream flow rate corresponding to a minimal efficiency can be identified for a certain dehumidification capacity *ε_L_*, when the ratio of the inlet absolute humidity of the process stream to the regeneration stream *χ* and the inlet temperature of the regeneration air stream *T_r,i_* are fixed; as *χ* increases, the value of *ε_L_* moves to lower values;
- The experimental operability of the system from Mohan et al. [32] exhibits limited cycle thermal performance;
- Higher cycle efficiency values can be achieved provided that the desiccant material is compatible with higher regeneration temperatures and the component structure, with lower flow rate ratio *μ*, lower sensible effectiveness *ε_s_*, and higher latent effectiveness *ε_L_*.

## Figures and Tables

**Table 1:** Dimensionless operative parameters.

$\mu =\frac{\overset{•}{m_{d,p}}}{{\overset{•}{m}}_{d,r}}$	$z=\frac{c_{pd,p}}{c_{pd,r}}$	$z_{v}=\frac{c_{pv,p}}{c_{pv,r}}$	$R_{v}=c_{pv,p}\left(1-\frac{1}{\gamma_{v}}\right)$
$\chi =\frac{Y_{r,i}}{Y_{p,i}}$	$z_{M}=\frac{c_{pv,p}}{c_{pd,p}}$	$R_{d}=c_{pd,p}\left(1-\frac{1}{\gamma_{d}}\right)$	$\gamma =\frac{c_{p}}{c_{V}}$

**Table 2:** Operative conditions of the experimental system of [32] and correspondingly calculated dimensionless parameters.

Mohan et al. [32]					
*m_d,r_*	0.3	kg/s	*m_d,p_*	0.2	kg/s
*Y_r,i_*	0.015	kg/kg*_a_*	*Y_p,i_*	0.008	kg/kg*_a_*
*T_r,i_*	43	^o^C	*T_p,i_*	11	^o^C
*T_r,o_*	46.80	^o^C	*T_p,o_*	15	^o^C
*Y_r,o_*	0.01633	kg/kg*_a_*	*Y_p,o_*	0.006	kg/kg*_a_*
*T_amb_*	30	^o^C	*χ*	1.875	-
*Y_amb_*	0.008	kg/kg*_a_*	*ε_s_*	0.125	-
*μ*	0.6667	-	*ε_L_*	0.2857	-
*z*	0.9977	-	*z_M_*	1.984	-
			*z_v_*	0.9902	-

## Nomenclature

COP	coefficient of performance
*c_p_*	specific heat at constant pressure, [J∙kg^−1^·K^−1^]
*c_V_*	specific heat at constant volume, [J∙kg^−1^·K^−1^]
*G*	dimensionless entropy generation
*ṁ*	mass flow rate, [kg∙s^−1^]
*R*	gas constant, [J∙mol^−1^·K^−1^]
*S*	entropy, [J∙K^−1^·s^−1^]
*s*	specific entropy, [J∙kg^−1^·K^−1^]
*T*	temperature, [K]
*Y*	air absolute humidity
*z*	dry air specific heat ratio
*z_v_*	vapour specific air ratio
*z_M_*	dry to vapour specific air ratio
Greek Symbol	
*ε*	effectiveness
*η*	cycle efficiency
*μ*	mass flow rate ratio
*γ*	specific heat ratio
*χ*	absolute humidity ratio
Subscripts	
*a*	absolute
*amb*	related to ambient
*d*	dry air
*gen*	irreversibility rate
*H*	high temperature
*i*	inlet value
*L*	latent/related to mass transfer
*min*	minimum
*o*	outlet value
*p*	related to process stream
*r*	related to regeneration stream
*R*	conditioned space (low temperature)
*s*	sensible
*TH*	thermal
*v*	related to vapour
